# Solitary juxtacortical lesion associated with anti-N-methyl-D-aspartate receptor encephalitis: a case report

**DOI:** 10.1186/s12883-020-01997-6

**Published:** 2020-11-20

**Authors:** Rupan Gao, Xiang Zhang, Abhijeet Kumar Bhekharee, Yue Zhang

**Affiliations:** 1 Department of Hematology, Zhongshan Hospital, Fudan University, Shanghai, China; 2 Department of Neurology, Huashan Hospital, Fudan University, No. 12 Mid Urumqi Road, Jin’an District, Shanghai, 200040 China; 3 Shanghai Medical College, Fudan University, Shanghai, China

**Keywords:** Seizure, Juxtacortical lesion, Demyelination, Electroencephalogram, Anti-N-methyl-D-aspartate receptor encephalitis

## Abstract

**Background:**

Anti-N-methyl-D-aspartate (NMDA) receptor encephalitis is a severe autoimmune encephalitis mediated by anti-NMDA receptor antibodies. Brain MRI manifestations vary and are non-specific. If there are any lesions, they tend to be diffusely or multifocally distributed. Solitary lesion is relatively rare.

**Case presentation:**

We report a 16-year-old girl who initially presented with focal seizures but developed severe psychiatric and extrapyramidal symptoms later on. Brain MRI revealed a solitary juxtacortical demyelinating lesion in the left frontal lobe. No enhancement was noted. Electroencephalogram captured epileptiform discharges in the same region. NMDAR IgGs were tested positive in the serum and cerebrospinal fluid. Corticosteroid and intravenous IgG were administered and the patient completely recovered. Brain MRI revealed a fainter lesion in the left frontal lobe.

**Conclusion:**

In very rare instances, anti-NMDA receptor encephalitis can present with a solitary brain lesion. A full panel of antibodies for autoimmune encephalitis is the key leading to the diagnosis.

## Background

Anti-N-methyl-D-aspartate (NMDA) receptor encephalitis was first described by Dalmau, et al., in 2007 [1]. Clinical picture covers a wide range of symptoms, including behavioral and psychiatric problems, memory loss, seizures, central hypoventilation, and movement disorders [1]. Brain MRI manifestations vary and are non-specific. More than half of the patients have normal MRI images [2]. If there are any brain lesions, they are more likely to be multifocal or diffuse [2, 3]. The most common finding on electroencephalogram (EEG) is diffuse slowing [4]. We herein report a case of anti-NMDA receptor encephalitis initially presenting with focal seizures and. a left frontal juxtacortical lesion after brain MRI.

## Case presentation

**Fig. 1:**
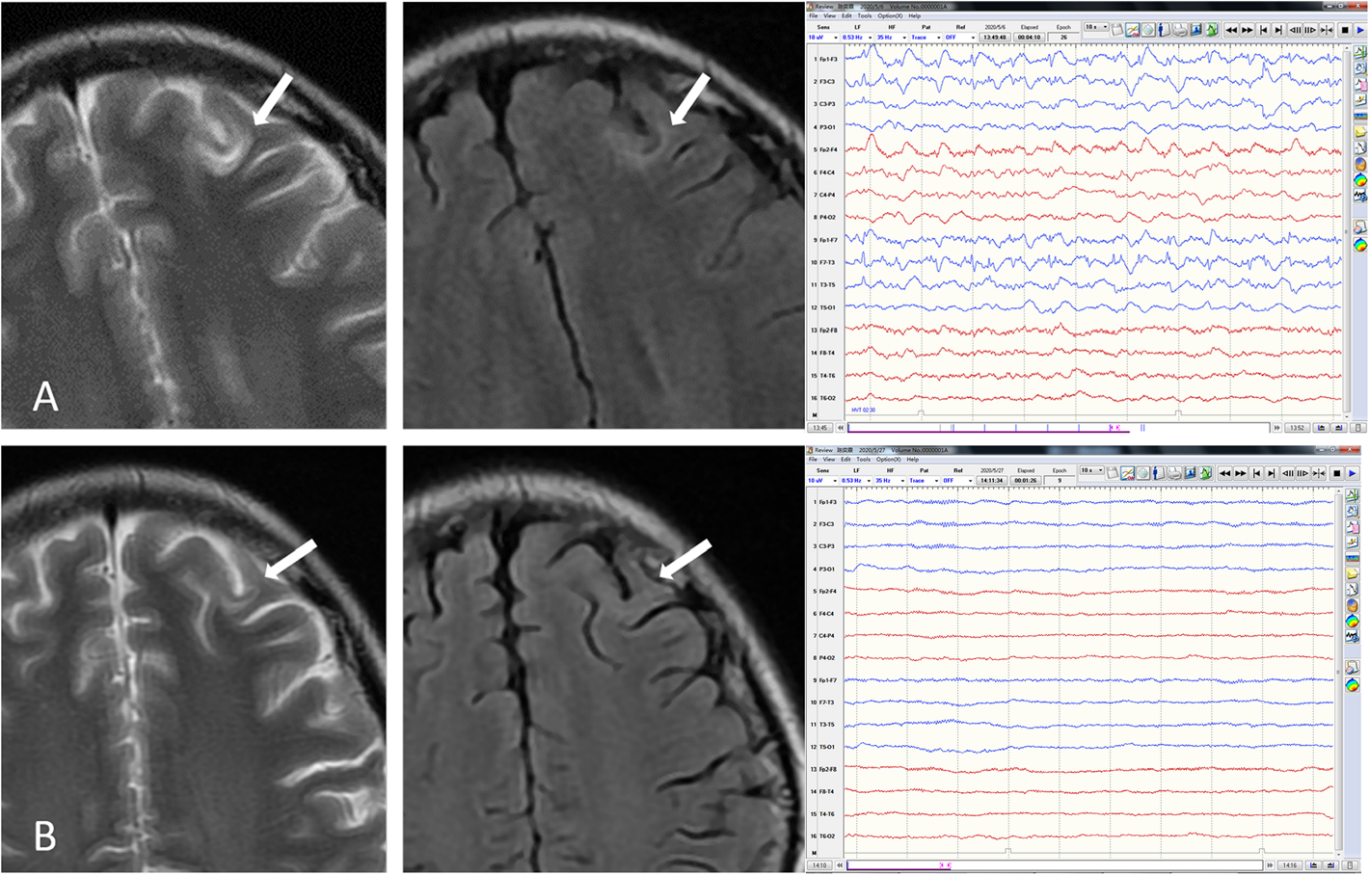
Brain MRI and EEG before and after treatment. Before treatment, T2WI and FLAIR (**a**, left and middle) showed a left frontal juxtacortical high signal intensity (upper arrows). EEG (**a**, right) revealed continuous 1.5 Hz spike wave complexes over the left frontal lobe. One month after admission, the lesion became faint on T2WI (**b**, left, arrow) and nearly undiscernible on FLAIR (B, middle, arrow). EEG (**b**, right) showed normal electrical activity

A 16-year-old girl presented to the emergency room with status epilepticus which was preceded by 10-day-long period of recurrent seizures. Ten days ago, she began to suffer from recurrent jerky movements in the right arm which sometimes evolved to generalized clonic-tonic seizures. Frequency and duration gradually increased despite the use of levetiracetam 500 mg Q12. Eventually, after the development of status epilepticus, she was sent to the emergency room where intravenous diazepam was injected. When she was transferred to the ward, she was sedated. Brain MRI revealed a left frontal unenhanced juxtacortical demyelinating lesion (Fig. 1 a, left and middle). EEG showed left frontal continuous epileptiform activities (Fig. 1a, right). Intravenous diazepam was tapered off and oral levetiracetam 1000 mg Q12 were prescribed. We also considered lesion resection if the seizures could not be controlled. On day 3, seizures stopped, but the patient was found to be irritable, aggressive and labile. Levetiracetam was replaced by oxcarbazepine 600 mg Q12 and Depakin 500 mg Q12 to reduce side effects. Nevertheless, her mental status continued to worsen. On day 7 she developed delirium, hallucination, delusion, insomnia, oromandibular dystonia, and bradykinesia.

On physical examination, she was agitated, mute and uncooperative. Neurological examination highlighted bradykinesia, nystagmus on lateral gaze, jaw-opening dystonia, difficulty in sticking out tongue, cogwheel rigidity in limbs and shuffling gait. Cerebrospinal fluid (CSF) analysis after lumbar puncture showed normal protein and glucose level but leukocytes were elevated to 17 × 10^6/L. Next generation sequencing for pathogen in CSF was unremarkable. NMDAR-antibody was positive in both serum and CSF samples (diluted 1:100) by cell-based assay (CBA). LGI1, CASPR2, AMPA1, AMPA2, GABAB, DPPX, IgLON5, AQP4, MOG and GFAP were all negative. CSF oligoclonal band was absent. Ovary teratoma was not detected by B-mode ultrasound. A repeat EEG showed diffuse beta waves without previous left frontal epileptiform activities. Anti-NMDA receptor encephalitis was considered. She was started on intravenous methylprednisone of 500 mg daily for 5 days and tapered. Two rounds of IV immunoglobulin (0.4 g/kg/d × 5 days) were used. Her mental status gradually stabilized. Furthermore, dystonia and bradykinesia decreased. On day 30, brain MRI revealed a faint left frontal juxtacortical lesion (Fig. 1b, left and middle). No other abnormalities were observed. EEG showed no epileptiform activities (Fig. 1b, right). On day 43, she was discharged with moderate dystonia. Thirty days after discharge, all the symptoms had resolved. She had no memory of the disease. The demyelinating lesion was even fainter on MRI and EEG showed normal result.

## Discussion and conclusion

Anti-NMDA receptor encephalitis is an autoimmune encephalitis with a wide range of symptoms. Common early symptoms include behavioral and speech problems, seizures, and abnormal movements [5]. While generalized seizures are common in female patients [6], focal seizures are more prevalent in male [6] and pediatric patients [7]. More than half of the patients have normal MR results [2, 8]. In those with brain lesions, hippocampus is the most commonly affected site with frontal and temporal lobes in the second and third places respectively [2]. Patients who developed lesions, had status epilepticus lasting 6 days on average [9], however, since the seizure of our patient was controlled within 24 h, her lesion could not be attributed to status epilepticus. What’s more, MRI lesions subsequently resolve in most patients [9]. Anti-NMDA receptor encephalitis may occasionally overlap with demyelinating syndromes. The demyelinating lesions tend to be multifocal or diffuse [3]. EEG abnormalities in anti-NMDA receptor encephalitis include diffuse slowing, epileptiform discharges, extreme delta brush, polymorphic delta rhythm, focal slowing and diffuse beta activities [4]. Diffuse slowing is the most common finding. Lateralized periodic discharges are uncommon [4].

The presence of right-sided focal seizures, solitary left frontal lesion and localized epileptiform discharges in our case highly suggested a focal process, such as focal cortical dysplasia or glioma. In this context, unnecessary surgical procedure is very likely to be performed. Rosenbloom MH, et al. reported a similar case of refractory seizures together with a focal enhancing lesion found on brain MRI, whereafter, the left middle frontal gyrus was surgically resected. Surgery did not bring any benefit [10]. Due to the fact that in the patient presented here seizures were controlled at early stage and psychiatric problems and movement disorders were exhibited later on, the patient did not undergo surgical procedure. Testing for anti-NMDAR antibodies in CSF and blood is the key to the correct diagnosis of anti-NMDAR encephalitis, but the false positiveness should be noted. Lu J, et al. reported a case of solitary cortical astrocytoma misdiagnosed as anti-NMDAR encephalitis due to positive NMDAR antibodies in serum and CSF. The only symptom was partial seizures in the right limb [11]. False positiveness of anti-NMDAR antibody in both serum and CSF was also reported in Lyme disease [12], False positiveness in serum has been found in multiple sclerosis [13], Creutzfeldt-Jakob disease [14], and schizophrenia [15].Therefore, besides autoantibodies detection, careful clinical observation is also warranted. False negative result is also possible. In a study by McCracken L et al., 65 serum and 32 CSF samples among 731 samples were indeterminate for antibodies with CBA. Re-evaluation with immunohistochemistry for reactivity to brain sections could further improve diagnostic accuracy [16]. As patients recover, their antibody titres in serum and CSF may decrease during monitoring, however, many patients remain antibody-positive even after recovery [17].

In conclusion, we have described an atypical case of anti-NMDA receptor encephalitis. Radiological and neuroelectrophysiological examinations impeded rather than facilitating the correct diagnosis. Testing CSF/blood for disease-associated autoantibodies is appropriate in patients (especially young people) with focal seizures, progressive psychiatric symptoms, emergent movement disorders and CSF pleocytosis.

## Data Availability

Not applicable.

## Abbreviations

NMDA: Anti-N-methyl-D-aspartate
MRI: Magnetic resonance imaging
EEG: Electroencephalogram
CSF: Cerebrospinal fluid
CBA: Cell-based assay
LGI1: Leucine-rich glioma inactivated 1
CASPR2: Contactin-associated protein-like 2
AMPA: α-amino-3-hydroxy-5-methyl-4-isoazolepropionic acid
GABAB: γ-Aminobutyric acid B
DPPX: Dipeptidyl-peptidase-like protein-6
AQP4: Aquaporin-4
MOG: Myelin oligodendrocyte glycoprotein
GFAP: Glial fibrillary acidic protein
